# S100a9 Protects Male Lupus-Prone NZBWF1 Mice From Disease Development

**DOI:** 10.3389/fimmu.2021.681503

**Published:** 2021-06-17

**Authors:** Laura M. Davison, Andres A. Alberto, Hardik A. Dand, Emma J. Keller, Madeline Patt, Ayesha Khan, Nina Dvorina, Alexandra White, Nodoka Sakurai, Lauren N. Liegl, Thomas Vogl, Trine N. Jorgensen

**Affiliations:** ^1^ Cleveland Clinic Lerner College of Medicine, Department of Molecular Medicine, Case Western Reserve University, Cleveland, OH, United States; ^2^ Department of Inflammation and Immunity, Lerner Research Institute, NE40, Cleveland Clinic Foundation, Cleveland, OH, United States; ^3^ Cleveland Clinic Lerner College of Medicine at Case Western Reserve University, Cleveland, OH, United States; ^4^ Institute of Immunology, University of Muenster, Muenster, Germany

**Keywords:** lupus, MDSC, S100A9, autoantibody, type I interferon, sex specific effects, mouse model, proteinuria

## Abstract

Systemic lupus erythematosus (SLE) is an autoimmune disorder disproportionally affecting women. A similar sex difference exists in the murine New Zealand Black/White hybrid model (NZBWF1) of SLE with all females, but only 30-40% of males, developing disease within the first year of life. Myeloid-derived suppressor cells (MDSCs) are prominent in NZBWF1 males and while depletion of these cells in males, but not females, promotes disease development, the mechanism of suppression remains unknown. S100a9, expressed by neutrophils and MDSCs, has previously been shown to exert immunosuppressive functions in cancer and inflammation. Here we investigated if S100a9 exerts immunosuppressive functions in NZBWF1 male and female mice. *S100a9* ^+/+^, *S100a9* ^+/-^ and *S100a9* ^-/-^ NZBWF1 mice were followed for disease development for up to 8 months of age. Serum autoantibody levels, splenomegaly, lymphocyte activation, glomerulonephritis and proteinuria were measured longitudinally or at the time of harvest. In accordance with an immunosuppressive function of MDSCs in male mice, *S100a9-*deficient male NZBWF1 mice developed accelerated autoimmunity as indicated by increased numbers of differentiated effector B and T cells, elevated serum autoantibody levels, increased immune-complex deposition and renal inflammation, and accelerated development of proteinuria. In contrast, female mice showed either no response to S100a9-deficiency or even a slight reduction in disease symptoms. Furthermore, male, but not female, *S100a9^-/-^* NZBWF1 mice displayed an elevated type I interferon-induced gene signature, suggesting that S100a9 may dampen a pathogenic type I interferon signal in male mice. Taken together, S100a9 exerts an immunosuppressive function in male NZBWF1 mice effectively moderating lupus-like disease development *via* inhibition of type I interferon production, lymphocyte activation, autoantibody production and the development of renal disease.

## Introduction

Systemic lupus erythematosus (SLE) is a chronic autoimmune inflammatory disease presenting with variable manifestations (1). Common indicators of SLE are elevated serum antinuclear autoantibodies (ANAs) and immune cell infiltration of affected organs such as the skin, heart, joints, or kidneys. The disease presents with a strong female predominance especially during child-bearing years, which has prompted much interest into the role of sex hormones (2–5). As such, numerous animal studies have shown that androgens exert a protective effect, while estrogens exacerbate disease development, the latter likely *via* direct effects on lymphocyte development and tolerance (6–12). Few studies however have looked into the mechanism driving immunoprotective effects of testosterone [reviewed in (13)]. Interestingly, immunosuppressive cells such as regulatory T and B cells, M2 macrophages and myeloid-derived suppressor cells (MDSCs) appear to be regulated by testosterone either directly during development or functionally during an immune response (14–20), suggesting that the presence of testosterone could lead to dysregulation of these cell populations and thus immune protection.

MDSCs have been ascribed a role in multiple immune disorders, but are best known for their role in cancer (21–25). The cells have a remarkable ability to suppress T cell responses by several methods including the production of reactive oxygen- and nitrogen-species (ROS, NOS), release of immunosuppressive cytokines (e.g. TGFβ, IL-10), production of indolamine 2’,3’-dioxygenase (IDO), and the production of S100A8/S100A9 heterodimers (also known as calprotectin) (26–29). While most of these effector mechanisms are shared with many other cell subsets, S100A8 and S100A9 proteins are exclusively expressed by myeloid cells constituting up to 45% of all cytosolic proteins in human neutrophils (30–32). Levels of heterodimers of S100A8 and S100A9 (S100A8/A9) are often elevated during inflammation, although whether the complex exerts immune stimulatory or immunoprotective properties depends on the underlying pathology (24, 25, 33–39). For example, *S100a9* ^-/-^ mice showed increased susceptibility to respiratory infection and increased renal fibrosis and damage supporting an immunoprotective or immune-resolution role (33, 34). Oppositely, S100a9-deficiency promoted reduced disease in animal models of arthritis and Alzheimer’s disease (24, 36). Importantly, none of these studies compared the response to S100a9-deficiency between males and females. In SLE patients (97% female cohort), serum levels of S100a9 have been found to be elevated and associated with SLEDAI scores and active bacterial infections (40). As for most studies, it remains unknown whether elevated S100a9 levels reflect proinflammatory or inflammation-resolution properties.

The (New Zealand black × New Zealand white)F1 (NZBWF1) mouse model develops a lupus-like disease characterized by hyperactive B cells, abnormal autoantibody production, glomerulonephritis, IgG immune complex (IC) deposition in the kidney glomeruli with complement fixation, and eventual renal failure resembling human SLE (41). Importantly, the model displays a similar female predominance with 100% of female NZBWF1 mice, but only 30-40% of male NZBWF1 mice, developing disease within one year. We and others previously reported an immunoprotective role for MDSC-like cells in male and young, prepubescent female NZBWF1 mice (14, 15, 42). While ROS/NOS has been identified as a mechanism of suppression in young females (14), the mechanism used by MDSCs in male NZBWF1 mice has not been identified. Based on studies suggesting a role for S100a9 in MDSC-driven immunosuppression in cancer (25), we hypothesized that MDSCs utilized S100a9 to inhibit immune activation and disease progression in NZBWF1 lupus-prone males. We report here that MDSC-like cells from *S100a9^-/-^* NZBWF1 mice failed to suppress B cell differentiation *in vitro*, suggesting that S100a9 is required for the immunosuppressive function of MDSC-like cells in these mice. Furthermore, male, but not female, *S100a9^-/-^* NZBWF1 mice showed increased disease development including elevated splenomegaly, hyperactive B and T cells, and accelerated renal disease. Interestingly, disease development in S100a9-deficient male mice was associated with elevated levels of type I interferon-stimulated gene transcripts and accumulation of IFNα-producing low density granulocytes (LDGs), suggesting that S100a9 limits type I interferon production in male lupus-prone mice hereby protecting the mice from disease development.

## Materials and Methods

### Animals and Cells

S100a9-deficient C57Bl/6 mice were obtained from Dr. Thomas Vogl (Westfälische Wilhelms-Universität Münster). S100a9-deficiency was backcrossed onto the NZB/BinJ and NZW/LacZ backgrounds for at least 8 generations. Crosses between NZB.*S100a9^+/-^* and NZW.*S100a9^+/-^* mice were performed to generate *S100a9^+/+^*, *S100a9^+/-^* and *S100a9^-/-^* NZBWF1 mice. For all analyses, littermates were used for comparison. Proteinuria was obtained from mice monthly starting at 3-4 months of age using dipsticks (Roche Biotech). Readings are presented on a scale from 0-4 (0, trace, 1, 2, 3, 4) corresponding with albumin levels of 0-400mg/dL urine as described by the manufacturer (Roche). When a mouse showed levels at or above 250 mg/dL (≥ 3), it was retested one week later. If consistently high, the mouse was euthanized for immediate analysis. Mice that did not develop proteinuria were euthanized at 8 months of age. For immunizations, *S100a9^+/+^*, *S100a9^+/-^* and *S100a9^-/-^* male and female NZBWF1 mice were immunized i.p. with 20µg (4-Hydroxy-3-nitrophenylacetyl)_27_ conjugated chicken γ-globulin (NP_27_-CGG) in complete Freund’s adjuvant (day 0). Immunized mice were bled on days -1, 7, 14, 21 and 28, and all animals were euthanized thereafter. Mice were maintained in the Biological Research Unit at the Lerner Research Institute, in accordance with Cleveland Clinic Foundation Animal Research Committee guidelines and all procedures were approved by the Institutional Animal Care and Use Committee of the Lerner Research Institute of the Cleveland Clinic Foundation and conducted in compliance with guidelines issued by the National Institutes of Health. For *in vitro* studies Gr1^high^CD11b^+^, Gr1^low^CD11b^+^, B220^high^CD19^+^CD138^-^ cells (*in vitro* differentiation assay) and Ly6C^high^CD11b^+^SSC^low^, Ly6C^low^CD11b^+^SSC^low^ cells (real-time RT-PCR) were isolated by fluorescence activated cell sorting (FACS) on a FACSAria I (BD Biosciences, San Jose, CA). All sorted cells were confirmed negative for CD3 and CD11c expression.

### Flow Cytometry

Spleen single cell suspensions were prepared by gently separating single cells between the frosted areas of two microscopy slides and red blood cells were lysed using 1x ACK buffer (0.15 M NH_4_Cl, 0.01 M KHCO_3_, 0.1 mM EDTA, pH 7.3). Single cells were incubated with unlabeled anti-CD16/32 antibodies in 1x phosphate buffered saline (PBS) for 20 min. after which fluorescently labeled antibodies specific for CD3, CD4, CD8, CD11b, CD11c, CD19, CD21/35, CD23, CD25, CD38, CD44, CD62L, CD69, B220, GL-7, Gr1, IgD, IgM, SiglecH (all from eBiosciences Inc., San Diego, CA), CD138 (BD Pharmingen, San Jose, CA), were added in combinations and samples were incubated for an additional 30 minutes. Flow cytometry was performed on a LSR Fortessa (BD Biosciences) and all analyses were performed using FlowJo version 9.7.5 or later (Tree Star Inc., San Carlos, CA).

### *In Vitro* B Cell Differentiation

B220^+^CD19^high^CD138^-^ B cells from *S100a9^+/+^* NZBWF1 male mice were plated (2 x 10^5^/well) with 5x10^4^ flow sorted Gr1^high^CD11b^+^ cells from either S100a9^+/+^ or S100a9^-/-^ NZBWF1 male mice in the presence or absence of recombinant IFNαA (500 units/ml, PBL InterferonSource, Piscataway, NJ) and recombinant CD40L (10µg/ml, EBiosciences) as previously described (14). After 72 hrs, 25,000 cells per well were transferred to ELISPOT plates (EMDMillipore, Burlington, MA) pre-coated with anti-Ig antibody (Southern Biotech, Birmingham, AL) for additional 20 hrs of culture. Plates were developed using HRP-conjugated anti-IgG and anti-IgM specific secondary antibodies (KPL, Milford, MA and Southern Biotech, respectively) and AEC Substrate set (BD Biosciences). The numbers of IgM and IgG-secreting cells were detected on an ELISPOT reader (CTL Immunospot, New York, NY) and number of spots per 10^5^ B cells were calculated.

### Immunohistochemistry

For histological analyses, kidneys were harvested at the end of the study (8 months of age) or earlier if mice presented with severe proteinuria. One half kidney was fixed in 10% formalin for at least 24 hours, transferred to 80% ethanol and embedded in paraffin. Five µm sections were cut and stained with hematoxylin/eosin (Newcomer Supply, Middleton, WI) or Masson’s Trichrome (Thermo Fisher Scientific, Waltham, MA) according to the manufacturer’s guidelines. Whole sections were scored in a blinded fashion by a renal pathologist (JN) at the Cleveland Clinic. Renal score: kidneys were evaluated on a scale of 0-5 for each of the following characteristics: mesangial hypercellularity, endocapillary hypercellularity, extracapillary proliferation (crescents), immune deposits, tubular atrophy, tubular casts, tubular dilation and interstitial fibrosis and inflammation for a maximum score of 40. 0 = absent, 1 = 1-5%, 2 = 6-10%, 3 = 11-20%, 4 = 21-50%, 5 > 50%. Glomerular area was calculated by measuring the area of 5-15 individual glomeruli per section for each mouse using the Keyence BZ-X analysis software (Keyence, Osaka, Osaka, Japan) and the Keyence BZ-X700 All-in-one microscope (Keyence, Osaka, Osaka, Japan). For identification of Mac2, S100a8 and S100a9, sections were blocked in 2% fetal bovine serum (FBS) in Hank’s Balanced Salt Solution (HBSS) for 30 min. Primary rat anti-mouse MAC2 (clone 125401)(BioLegend), rat anti-mouse S100a8 (clone MAB3059) or mouse S100A9 clone AF2065) (both from (R & D Systems, Minneapolis, MN) antibodies were added at 1:1500, 1:250 and 1:250 dilution, respectively, and sections were left to incubate for 1 hour in a humidified chamber at room temperature. After washing with 1x PBS, slides were applied rat-on-mouse horse radish peroxidase (HRP) polymer (#RT517) (Biocare medical, Pacheco, CA) for 20 minutes at room temperature, washed with 1x PBS, after which 3,3’-diaminobenzidine (DAB) substrate was added for 1 minute. Slides were counterstained with hematoxylin 7211 (Thermo Fisher Scientific) for 1 minute, treated with Clear Rite™ and mounted for microscopy. All light microscopy images were taken on an Eclipse 55i Nikon microscope equipped with a 12 megapixel DS-Ri1 Digital Camera.

### Immunofluorescence Staining

Half kidneys and 2mm cross sections of spleens were isolated and immediately frozen in OCT™. Five µm sections were cut and sections were stained for the presence of IgG and complement C’3, or B220 and GL7, respectively. Briefly, sections were fixed with cold acetone and blocked with unlabeled anti-mouse CD16/CD32 (1:200, EBiosciences) in 10% non-immune goat serum (Invitrogen). Texas-red conjugated anti-mouse IgG (1:500, Southern Biotech), FITC-conjugated anti-mouse C’3 (1:500, ICL), FITC-conjugated anti-B220 antibodies (EBiosciences), biotinylated anti-GL7 antibodies (eBioscience), and Alexa Fluor 568–conjugated streptavidin (Invitrogen) were added as indicated for each staining combination and sections were incubated overnight at room temperature. The next day, sections were washed and mounted with 70% glycerol. Imaging was done on a Keyence BZ-X700 All-in-one microscope (Keyence, Osaka, Japan) and images were quantified using the Keyence BZ-X analysis software (Keyence, Osaka, Japan). Colocalization of IgG/C’3 in renal samples is displayed by the color yellow.

### Hep2-Assay

The Hep2 assay was performed according to the manufacturer’s guidelines (Bio-Rad, Hercules, CA). Positive and negative controls provided with the manufacturers’ kit were added (1:64 dilution) to two wells on each slide. Serum from *S100a9* ^+/+^, *S100a9* ^+/-^ and *S100a9* ^-/-^ mice were diluted 1:10 in 1x phosphate buffered saline and added to remaining wells. Images were obtained on a Leica Leitz fluorescence microscope and processed using Image-Pro Plus software.

### ELISA

Serum anti-chromatin and anti-histone IgG levels were determined as previously described using serum diluted 1:300 in serum diluent (43). Briefly, microtiter plates (Immulon 2HD, Thermo Fisher Scientific) were coated with purified chromatin or total histones overnight at 4°C, blocked in 5% gelatin/PBS for >2 hours, and incubated with diluted serum samples for 2 hours. Secondary horseradish peroxidase–conjugated anti-mouse IgG antibodies (Invitrogen, Calsbad, CA) were added for 1.5 hours, and the plates were developed using 10 mg/ml 2,2’-Azinobis [3-ethylbenzothiazoline-6-sulfonic acid (ABTS) in McIlwain’s buffer (0.09*M* Na2HPO4, 0.06*M* citric acid, pH 4.6) or a 3,3′,5,5′-Tetramethylbenzidine (TMB) Substrate Kit (Thermo Fisher Scientific). Anti-dsDNA IgG levels were determined using serum diluted 1:100 according to the manufacturer’s guidelines (Alpha Diagnostics, Santa Monica, CA). Total serum IgG and total serum IgM levels were determined on serum diluted 1:100,000 as previously described (43). Serum S100a8/a9 heterodimer was detected using the mouse S100a8/S100a9 ELISA kit on serum diluted 2-4 times (MyBiosource, CA, USA). IgG1 specific to 4-Hydroxy-3-nitrophenylacetyl-chicken gamma globulin (NP-CGG) was determined as previously described using serum diluted 1:10,000 (42). Serum levels of soluble RAGE (sRAGE) and HMGB1 were performed on 1:10 diluted serum from 4 month old mice according to the manufacturer’s instructions (MyBiosource). Serum BAFF levels were determined on 1:3-1:12 diluted serum samples according to the manufacturer’s instructions (R&D Systems). All samples were run in duplicate. All ELISA plates were read on a Victor™ plate reader (Perkin Elmer) at 405nm or 450 nm. When possible, concentrations were calculated based on standard curves provided with the individual kits. All analyses were done using GraphPad Prism v. 5.02 (San Diego, CA).

### Real Time RT-PCR

For detection of *S100a4*, *S100a8* and *S100a9* transcript levels Gr1^high^CD11b^+^ cells were flow sorted from male and female 4 week old NZBWF1 mice. RNA was isolated using the Micro RNeasy kit (Qiagen, Germantown, MD) and cDNA was prepared using qScript cDNA Supermix (QuantaBio, Beverly, MA). Transcript levels were determined in a SyBr-green based assay (PerfecCTa ^®^ SYBR ^®^ Green Fastmix ^®^ ROX (QuantaBio)) using the following primers: *S100a4* Forward 5’-ctactgaccagggagctg c-3’, *S100a4* Reverse 5’-tgttgctgtccaagttgctca-3’, *S100a8* Forward 5’-gagtgtcctcagtttgtgcagaa-3’, *S100a8* Reverse 5’-tgagatgccacacccactttt-3’, *S100a9* Forward 5’-gaagcacagttggcaacctt-3’, *S100a9* Reverse 5’-caggtcctccatgatgtcat-3’, *β2M* Forward 5’-tcagtcgcggtcgcttc-3’, *β2M* Reverse 5’-caagcaccagaaagactagggtc-3’. For detection of *Irf7*, *Isg15*, *Ifi202*, and *Ifna* total splenocytes from 8 months old *S100a9^+/+^* and *S100a9^-/-^* NZBWF1 male and female mice were isolated, RNA extracted and cDNA prepared as described above. βeta 2-microglubulin (*β2M*) was used as the internal control gene and the following gene specific primers were used: *Irf7* Forward 5’-gcgtaccctggaagcatt tc-3’, *Irf7* Reverse 5’-gcacagcggaagttggtct-3’, *Isg15* Forward 5’-ggtgtccgtgactaactccat-3’, *Isg15* Reverse 5’- tggaaagggtaagaccgt cct-3’, *Ifi202* Forward 5’-caagcctctcctggacctaa-3’, *Ifi202* Reverse 5’-ctaggatgccactgctgttg-3’, *Pan-Ifna* Forward 5’-cttccacaggatcactgtgta cct-3’, *Pan-Ifna* Reverse 5’-ttctgctctgaccacctccc-3’.

### Statistical Analyses

GraphPad Prism was used for all statistical analyses. One-way ANOVA tests were done for all group comparisons. One-way ANOVA results are provided in figure legends. The Kruskal-Wallis test followed by Dunn’s multiple comparisons test was done for one-time point proteinuria scores (non-Gaussian distribution). Longitudinal proteinuria data were evaluated using a log rank test. Student’s t-test with Welch’s correction was done for pair-wise comparisons. p < 0.05 was considered statistically significant for all analyses.

## Results

### *S100a9* mRNA Expression Is Elevated in Gr1^high^CD11b^+^ Cells From Male NZBWF1 Mice

**Figure 1:**
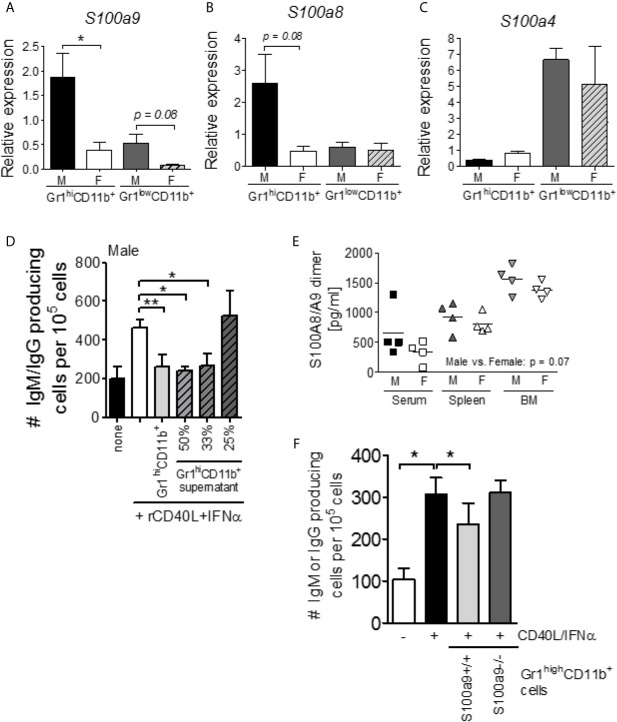
*S100a9* expression is elevated within Gr1^high^CD11b^+^ cells of male lupus-prone mice and regulates B cell differentiation *in vitro* and antibody production *in vivo*. Gr1^high^CD11b^+^ and Gr1^low^CD11b^+^ cells were isolated from 9 week old male and female NZBWF1 lupus-prone mice by flow cytometry and levels of *S100a9* **(A)**, *S100a8* **(B)**, and *S100a4* **(C)** mRNA were determined by RT-PCR analysis (*n* = 4). Graphs show mean +/- SEM. **(D)** Supernatants from overnight cultured Gr1^high^CD11b^+^ cells from 4 wk old NZBWF1 male mice suppress IFNα/CD40L-driven B cell differentiation. Shown is the average (± SEM) of 4 independent experiments. **(E)** S100A8/A9 heterodimer levels in serum, spleen and bone marrow from 9 wk old NZBWF1 mice. Each symbol represents one mouse. Male vs. female: p = 0.07; two-way ANOVA. **(F)** Gr1^high^CD11b^+^ cells from 9 wk old *S100a9*-deficienct male NZBWF1 mice fail to suppress cytokine-driven B cell differentiation *in vitro*. IgM/IgG-secreting cells were enumerated by ELISPOT and are presented as number of cells per 10^5^ plated B cells. Data shown represent the mean ± SEM of 4 independent assays. *P < 0.05; **P < 0.01, Student’s unpaired *t* test.

Gr1^high^CD11b^+^ MDSC-like cells exert immunosuppressive functions and are increased in male NZBWF1 mice as compared to female littermates (14). We have previously shown that *female* Gr1^high^CD11b^+^ cells from 4 week old NZBWF1 mice utilized ROS/NOS production to inhibit cytokine-driven B cell differentiation, but failed to identify the mechanism used by *male* NZBWF1-derived cells (14). S100a8/a9 heterodimers are produced predominantly by neutrophils (30, 31) and have previously been associated with immunosuppression (25). We isolated Gr1^high^CD11b^+^ and Gr1^low^CD11b^+^ cells from 9 week old male and female NZBWF1 lupus-prone mice and determined levels of *S100a4*, *S100a8*, and *S100a9* mRNA. *S100a9* mRNA levels were significantly elevated in male Gr1^high^CD11b^+^ cells as compared to female-derived cells (p < 0.05) (**Figure 1A**). A similar trend was seen in Gr1^low^CD11b^+^ cells (p = 0.08). Levels of *S100a8* mRNA were also elevated in male Gr1^high^CD11b^+^ cells as compared to female-derived cells (p = 0.08) (**Figure 1B**), while levels of *S100a4* mRNA were unchanged between males and females (**Figure 1C**).

To investigate whether the previously reported mechanism of suppression by male Gr1^high^CD11b^+^ cells depended on a secreted factor, we cultured flow sorted NZBWF1 male CD19^+^CD138^-^ B cells with supernatants from overnight cultured flow-sorted male Gr1^high^CD11b^+^ cells in the presence of differentiation-inducing cytokines (CD40L and IFNα). B cell differentiation was effectively blocked in a dose-dependent manner (**Figure 1D**), suggesting that the inhibitory factor was indeed secreted by Gr1^high^CD11b^+^ cells. As S100a8/S100a9 heterodimers can be secreted by neutrophils, we evaluated levels of S100a8/S100a9 heterodimers in serum, spleen, and bone marrow samples. S100a8/S100a9 heterodimers were slightly increased in male NZBWF1 mice at all sites (p = 0.07) (**Figure 1E**), prompting us to further investigate a possible role for S100a9 in Gr1^high^CD11b^+^ MDSC-like cell-mediated immunosuppression during lupus-like disease development *via* the generation of S100a9-deficient NZBWF1 (see **Supplemental Figure 1**). Gr1^high^CD11b^+^ cells were sort purified from *S100a9^+/+^ and S100a9^-/-^* male NZBWF1 mice and tested for their ability to suppress B cell differentiation *in vitro* as previously described (14). Supporting an immunosuppressive role, S100a9-sufficient, but not S100a9-deficient, male Gr1^high^CD11b^+^ cells inhibited B cell differentiation *in vitro* (**Figure 1F**).

### Elevated Antibody Responses in Male *S100a9^-/-^* NZBWF1 Mice

**Figure 2:**
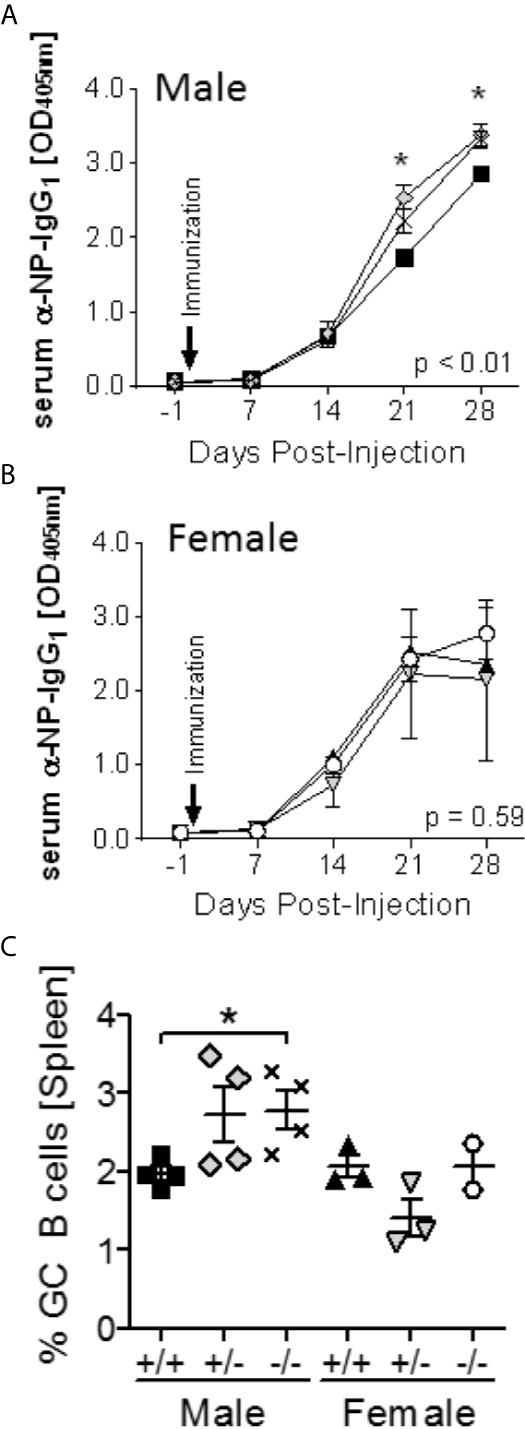
Enhanced response to T-dependent Ag immunization in male *S100a9^-/-^* NZBWF1 mice. Male **(A)** *S100a9^+/+^* (black squares), *S100a9* ^+/-^ (grey diamonds), *S100a9^-/-^* (symbol X), and female **(B)** *S100a9^+/+^* (black triangles, up), *S100a9* ^+/-^ (grey triangles, down), *S100a9^-/-^* (open circles) NZBWF1 mice (*n* = 3-4) were immunized on day 0 with NP_27_-CGG in CFA. Levels of NP-specific IgG1 antibodies were determined on days -1, 7, 14, 21 and 28. *p < 0.05, two-way ANOVA. **(C)** GC B cells were identified by flow cytometry four weeks post immunization. *p < 0.05, Student’s unpaired *t* test with Welch’s correction. One-way ANOVA: p < 0.05.

To determine the *in vivo* effect of S100a9-deficiency, *S100a9* ^+/+^, *S100a9* ^+/-^ and *S100a9* ^-/-^ NZBWF1 mice were immunized with NP-CGG in CFA, and serum evaluated for the levels of NP-specific IgG_1_ levels. Supporting an immunosuppressive role *in vivo*, Male *S100a9^-/-^* and *S100a9^+/-^* mice displayed significantly increased anti-NP IgG_1_ levels as compared to male *S100a9^+/+^* mice (p < 0.01; **Figure 2A**). In contrast, immunization of *S100a9^+/+^*, *S100a9* ^+/-^ and *S100a9* ^-/-^ female NZBWF1 mice resulted in similar levels of NP-specific antibodies between the strains (**Figure 2B**), suggesting a sex-specific effect of S100a9 in NZBWF1 lupus-prone mice. Four weeks post immunization, animals were euthanized and levels of splenic germinal center (GC) B cells and T follicular helper (Tfh) cells were analyzed. Further supporting a male-specific effect, percentages of GC B cells were significantly elevated in male, but not female, *S100a9^-/-^* NZBWF1 mice (**Figure 2C**). In contrast, the frequencies of Tfh cells were unchanged between all the groups, likely reflecting the advanced time point of evaluation (data not shown).

### Male *S100a9^-/-^* NZBWF1 Mice Develop Splenomegaly And Present With Elevated Anti-Nuclear Autoantibodies

**Figure 3:**
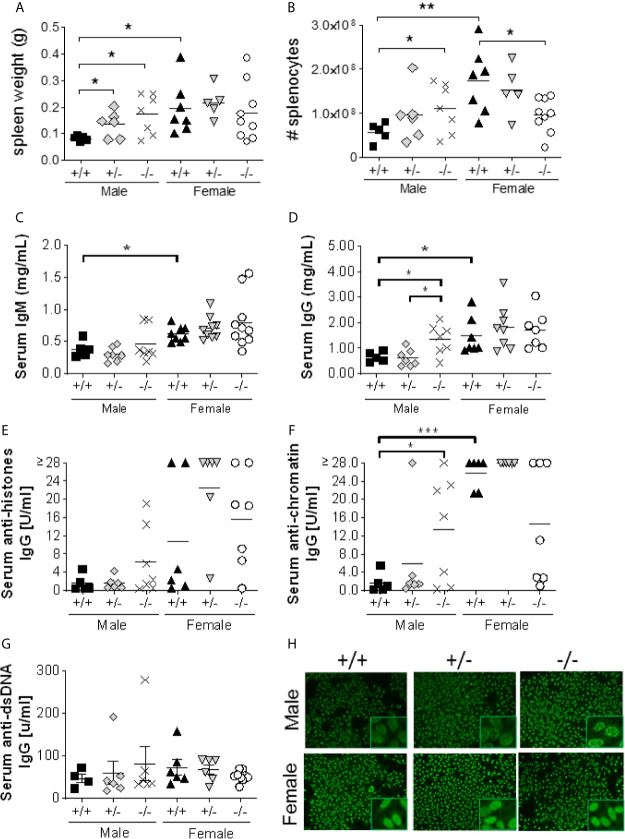
*S100a9^-/-^* male BWF1 mice develop splenomegaly and elevated anti-nuclear autoantibodies. Spleens were harvested from eight month old BWF1 lupus-prone mice. Spleen weight **(A)** and splenocyte count **(B)** were measured (*n* = 5-11). Each symbol represents one individual mouse, horizontal lines represent mean values. C-G) Serum was obtained from five months old BWF1 lupus-prone mice and serum autoantibody levels were determined by ELISA: serum IgM **(C)**, serum IgG **(D)**, anti-histone IgG **(E)**, anti-chromatin IgG **(F)**, anti-dsDNA **(G)**. Each symbol represents one individual mouse (*n* = 5-11). **(H)** Hep2 Assay depicting nuclear and perinuclear antibody staining. Representative pictures shown from 5-11 mice/group. *p < 0.05; **p < 0.01; ***p < 0.001, Student’s unpaired *t* test with Welch’s correction. One-way ANOVA: p = 0.21 **(A)**, p < 0.05 **(B)**, p < 0.01 **(C)**, p < 0.01 **(D)**, p < 0.01 **(E)**, p < 0.0001 **(F)**, p = 0.87 **(G)**.

Splenomegaly is a well-established indicator of mouse lupus-like disease. Male *S100a9^-/-^* and *S100a9* ^+/-^ NZBWF1 mice displayed significantly larger spleens than male *S100a9^+/+^* mice (p < 0.05, **Figure 3A**). In contrast, no significant difference was observed between S100A9-sufficient and S100A9-deficient female mice. A similar trend was observed for total splenocyte count, although this measure only reached statistical significance when comparing *S100a9^-/-^* and *S100a9^+/+^* male mice (p < 0.05, **Figure 3B**). As expected, S100a9-sufficient female mice exhibited significantly larger spleens than S100a9-sufficient male mice (p < 0.05-0.01, **Figures 3A, B**) and *S100a9^-/-^* females (p < 0.05), suggesting that S100a9 might play a different role in disease progression in older female NZBWF1 mice.

We hypothesized that S100a9-deficient male, but not female, NZBWF1 mice would present with elevated levels of serum anti-nuclear autoantibodies; a hallmark of lupus-like disease in this animal model. Cohorts of male and female *S100a9^+/+^*, *S100a9^+/-^*, *and S100a9^-/-^* NZBWF1 mice were followed for up to 8 months of age or until severe renal disease was present (*n* = 5-11). Serum was obtained monthly and tested for total IgG, total IgM, and anti-nuclear autoantibodies by ELISA. At 4-5 months of age, serum IgM levels were significantly increased in all female mice as compared to male mice (p < 0.05), but there was no major difference between mice of similar sex (**Figure 3C**). In contrast, serum total IgG was significantly elevated in male *S100a9* ^-/-^ NZBWF1 mice as compared to *S100a9* ^+/-^ and *S100a9* ^+/+^ male littermates (p < 0.05, **Figure 3D**), while there was no difference between female mice. We also observed a modest elevation in serum anti-histones IgG and serum anti-dsDNA IgG levels and a significant increase in anti-chromatin IgG levels in male *S100a9^-/-^* mice (p < 0.05), with concentrations approaching those measured in the female cohorts (**Figures 3E–G**). Interestingly, there was a trend towards less serum anti-chromatin IgG in female *S100a9* ^-/-^ mice as compared with female *S100a9* ^+/+^ mice (**Figure 3F**, p = 0.07). Further analysis of the nature of serum autoantibodies from *S100a9* ^+/+^, *S100a9* ^+/-^ and *S100a9* ^-/-^ male and female NZBWF1 mice supported stronger and more wide-spread recognition of both cytoplasmic and nuclear antigens by serum antibodies from male *S100a9* ^-/-^ mice as compared with antibodies from male *S100a9* ^+/+^ mice, but no difference between female *S100a9* ^+/+^, *S100a9* ^+/-^ and *S100a9* ^-/-^ mice (**Figure 3H**).

### *S100a9^-/-^* Male NZBWF1 Mice Develop Spontaneous B Cell Hyper-Activation

**Figure 4:**
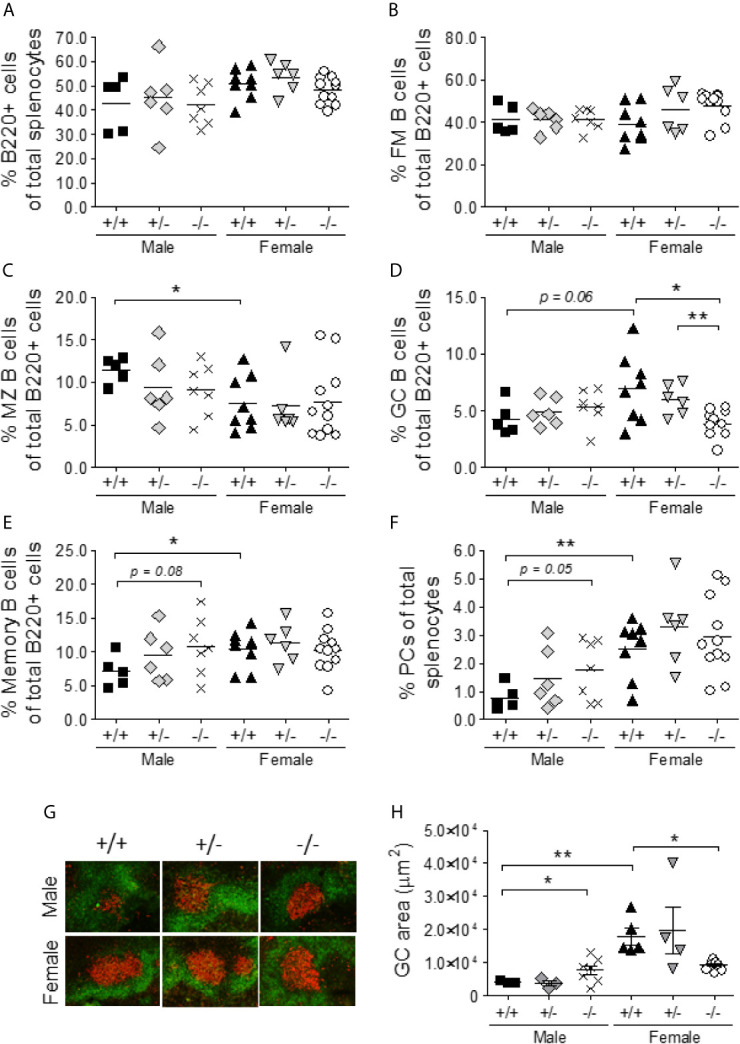
*S100a9-*deficiency results in increased spontaneous germinal reactions in male BWF1 mice. Spleens were harvested from eight month old BWF1 lupus-prone mice and percentages of B cell subsets were determined by flow cytometry: **(A)** total B220+ cells, **(B)** Follicular mature (B220^+^CD23^high^CD21^low^IgM^low^) B cells, **(C)** Marginal Zone B cells (B220^+^CD21^high^CD23^low^ IgM^high^), **(D)** Germinal center B cells (B220^+^GL7^+^CD38^low^IgM^low^), **(E)** memory B cells (B220^+^ CD38^high^GL7^-^IgM^-^) and **(F)** Plasma cells (B220^-/low^CD138^+^IgM^-^IgD^-^). G) Frozen spleen sections were stained for B cells (B220-FITC) and germinal centers (GL-7-TexasRed). Representative pictures are shown. H) Germinal center areas from **(G)** were determined using the Keyence BZ-X analysis software. **(A–F, H)** Each symbol represents one individual mouse (*n* = 5-11). *p < 0.05; **p < 0.01, Student’s unpaired *t* test with Welch’s correction. One-way ANOVA: p = 0.13 **(A)**, p = 0.14 **(B)**, p = 0.33 **(C)**, p < 0.01 **(D)**, p = 0.36 **(E)**, p < 0.01 **(F)**, p < 0.01 **(H)**.

Lupus-like disease in NZBWF1 mice is characterized by hyperactive B cells, abnormal germinal center (GC) formation, and an accumulation of memory B cells and plasma cells (44, 45). Despite the difference in spleen size, there was no difference in the percentages of total B220^+^ cells and follicular mature B cells (CD23^high^CD21^low^IgM^low^) in the spleens of male and female S100a9-deficient and -sufficient NZBWF1 mice (**Figures 4A, B**). In alignment with previous findings (46), female *S100a9* ^+/+^ NZBWF1 mice displayed reduced percentages of MZ B cells (B220^+^CD21^high^CD23^low^) as compared with *S100a9* ^+/+^ male mice, however a similar pattern was not observed in male *S100a9* ^-/-^ mice (**Figure 4C**). As expected, the percentage of GC B cells was increased in female *S100a9* ^+/+^ mice as compared to male *S100a9^+/+^* mice (p = 0.057), but there was no significant difference in the percentages of GC B cells between S100a9-sufficient and -deficient male mice (**Figure 4D**), despite a significant increase in GC area (p < 0.05) (**Figures 4G, H**). Female *S100a9* ^-/-^ mice showed a decreased frequency of GC B cells as compared with female *S100a9* ^+/+^ mice (p < 0.05)(**Figure 4D**), a pattern that was mimicked by smaller size GCs in female S100a9^-/-^ mice (**Figures 4E, F**). Finally, frequencies of memory B cells and plasma cells (PCs) were elevated in male *S100a9^-/-^* mice as compared with male *S100a9* ^+/+^ mice (p = 0.08 and p = 0.05, respectively) and approached levels observed in *S100a9^+/+^* female mice (**Figures 4G, H**). Taken together, several B cell subsets, plasma cells and GCs from male *S100a9^-/-^* NZBWF1 mice acquired an intra-splenic balance approaching that observed in female *S100a9* ^+/+^ NZBWF1 mice, supporting a role for S100a9 in the regulation of B cell differentiation in male NZBWF1 mice.

### S100A9-Deficiency Drives Spontaneous T Cell Activation and Differentiation in Male NZBWF1 Mice

**Figure 5:**
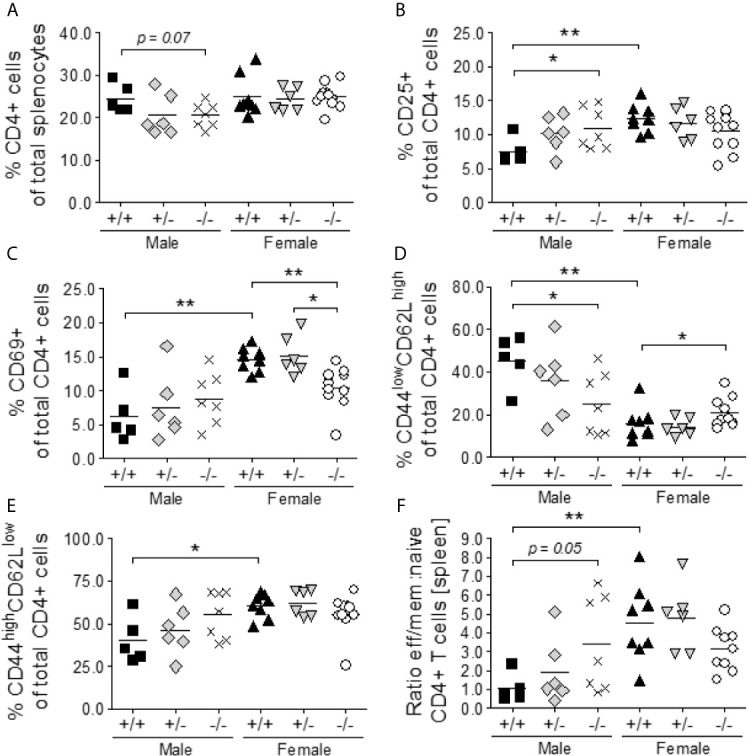
*S100a9-*deficiency promotes the activation and differentiation of CD4+ T naïve cells to effector memory cells in *S100a9^-/^* ^-^ male BWF1 mice. Spleens were harvested from eight month old BWF1 lupus-prone mice and percentages of T lymphocytes and their subsets were determined by flow cytometry: total CD4^+^ cells **(A)**, CD25^+^ CD4^+^ cells **(B)**, CD69^+^ CD4^+^ cells **(C)**, naïve (CD44^low^CD62L^high^) CD4^+^ cells **(D)**, effector memory (CD44^high^CD62L^low^) CD4^+^ cells **(E)**, and the ratio between naïve and effector memory cells **(F)**. Each symbol represents one individual mouse. *p < 0.05; **p < 0.01, Student’s unpaired *t* test with Welch’s correction. One-way ANOVA: p < 0.05 **(A)**, p < 0.05 **(B)**, p < 0.0001 **(C)**, < 0.0001 **(D)**, p < 0.05 **(E)**, p < 0.01 **(F)**.

It has previously been described that B cell hyper-activation and autoantibody production in female NZBWF1 mice depends on T cell help (47). We determined the percentages of T helper cell subsets in S100a9-sufficient and -deficient, male and female mice. There was no statistically significant difference in the percentages of total CD4^+^ T cells among the cohorts of male and female mice (**Figure 5A**). The expression of CD25 and CD69, was significantly elevated on female *S100a9^+/+^* CD4^+^ cells as compared to male *S100a9^+/+^* CD4^+^ cells (p<0.01), while male *S100a9* ^-/-^ NZBWF1 mice displayed elevated percentages of CD25^+^CD4^+^ cells, but not CD69^+^CD4^+^ cells, as compared with male *S100a9* ^+/+^ NZBWF1 mice (p < 0.05) (**Figures 5B, C**). Interestingly, we again observed a partial normalization in the levels of CD69^+^CD4^+^ T cells in *S100a9^-/-^* female mice as these approached levels in *S100a9^+/+^* males, but no significant change in the frequency of CD25^+^CD4^+^ T cells between female mice (**Figures 5B, C**).

Finally, male *S100a9^+/+^* NZBWF1 mice expressed high levels of naïve CD4^+^ T cells (CD62L^high^CD44^low^) and low levels of effector/memory CD4^+^ T cells (CD62L^low^CD44^high^), while the balance was shifted in both male *S100a9* ^-/-^ and female *S100a9* ^+/+^ NZBWF1 mice (p = 0.05 and p < 0.01, respectively) (**Figures 5D–F**). Thus, male *S100a9^-/-^* NZBWF1 mice presented with a hyperactive T cell phenotype similar to that of female *S100a9^+/+^* NZBWF1 mice, further supporting an immunosuppressive role for S100a9 in male NZBWF1 mice.

### *S100a9^-/-^* Male NZBWF1 Mice Develop Enhanced Glomerulonephritis and Renal Damage

**Figure 6:**
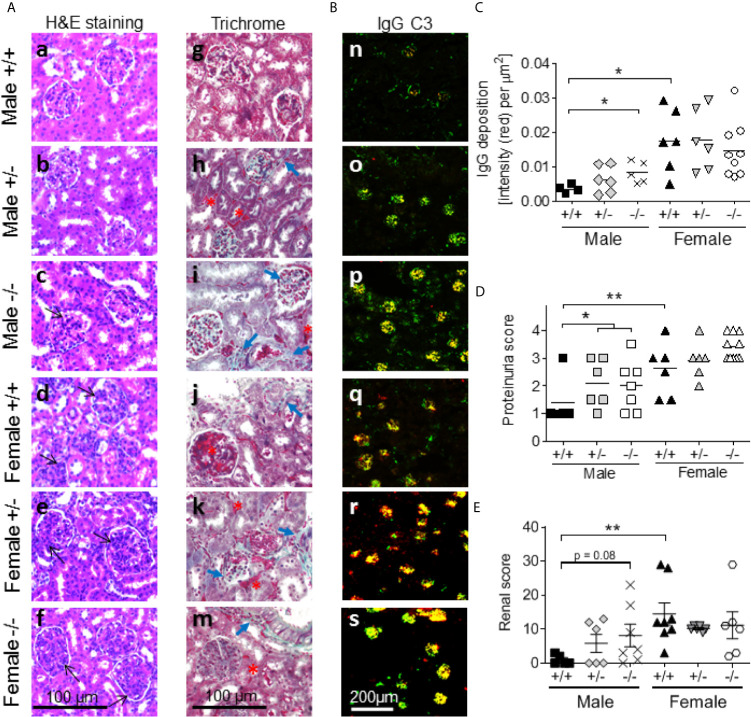
S100a9-deficiency promotes proteinuria and renal damage in male BWF1 mice. Kidneys were harvested from eight month old BWF1 lupus-prone mice (*n* = 5-11) and stained. **(A)** H&E (a-f) and Masson’s Trichrome (g-m) were performed to assess glomerulonephritis, mesangial proliferation (black open-head arrows), interstitial inflammation, tubular atrophy, collagen deposits (blue closed-head arrows), and cast formation (red asterisk). All images were taken at similar settings and magnifications. **(B, C)** Deposition of IgG-immune complexes (red) and fixation of complement factor 3 (green) were determined by immunofluorescent staining of kidneys (**B**, n-s). Colocalization of IgG and C’3 can be seen as yellow. IgG deposition was quantified **(C)**. **(D)** Proteinuria readings at the time of harvest. **(E)** Renal histology scores. Each symbol represents one individual mouse. *p < 0.05; **p < 0.01, Student’s unpaired *t* test with Welch’s correction **(C, D)**, Student’s paired t- test **(E)**. One-way ANOVA: p < 0.01 **(C)**, p < 0.01 **(D)** and p < 0.01 **(E)**.

Lupus-like renal disease in female NZBWF1 mice is characterized by the development of glomerulonephritis, the deposition of IgG-immune complexes (IgG-IC) in the glomeruli, complement fixation, and renal damage (44, 48). Kidneys were harvested from all mice at the end of the study and evaluated by immunostainings. Male *S100a9^+/+^* mice appeared relatively healthy at eight months of age with small glomeruli and minimal inflammation (**Figure 6A, **panels a, g). In contrast, *S100a9* ^+/-^ and *S100a9* ^-/-^ male mice displayed a progressive worsening as characterized by increased mesangial proliferation (black open-head arrows) and interstitial fibrosis (**Figure 6A, **panels b, c), increased collagen deposits (closed-head blue arrows), tubular atrophy, and visible proteinaceous material (black asterisk) (**Figure 6A,** panels h, i). Irrespective of their genotype, all females displayed significantly more damaged kidneys with significantly increased mesangial cell proliferation, interstitial fibrosis, and tubular casts than their male counterparts (**Figure 6A,** panels d-f and j-m).

Kidneys were further evaluated for the presence of IgG-IC deposition and complement factor C’3 fixation by immunostaining. *S100a9^-/-^* males displayed increased levels of IgG-IC deposition (red color), increased C’3 fixation to the glomeruli (green color), and expanded areas of co-localization with C’3 (yellow color) as compared with *S100a9^+/+^* males (**Figure 6B,** panels n-p). In contrast, all female mice showed comprehensive overlapping IgG-IC deposition and complement C’3 fixation (**Figure 6B,** panels q-s, yellow color). Quantification of IgG-IC deposition showed statistically more depositions in *S100a9^-/-^* males and *S100a9^+/+^* females as compared with *S100a9^+/+^* males (p < 0.05 and p < 0.01, respectively) (**Figure 6C**). As renal damage progresses to renal failure in female NZBWF1 mice, proteinuria levels were measured monthly in all animals starting at 3-4 months of age to monitor the onset of renal failure. At 8 months of age, levels of proteinuria were significantly increased in male *S100a9^-/-^* and *S100a9^+/-^* NZBWF1 mice as compared to *S100a9^+/+^* male littermates (p < 0.05), while there was no statistical difference between the female mice (**Figure 6D**). The difference among male mice was not apparent at earlier time points (**Supplemental Figure 2A**). There was also no difference in the average time for disease to develop among female mice: *S100a9^+/+^*: t = 6.8 months (n = 5), *S100a9^+/-^*: t = 6.8 months (n = 5), and *S100a9^-/-^*: t = 6.875 months (n = 8) months, indicating that S100a9-deficiency did not accelerate disease development in the females (**Supplemental Figure 2A**). Finally, renal damage was supported by an elevated renal score in female *S100a9^+/+^* mice (p < 0.01), and a trend towards a higher score in male *S100a9* ^-/-^ mice (p = 0.08). There was no statistically significant difference among the groups of female mice (**Figure 6E**). Given the presence of areas of fibrosis, we analyzed kidney sections for levels of pro-inflammatory Mac2^+^ macrophages. Numbers of Mac2^+^ macrophages within glomeruli increased slightly in both *S100a9^+/-^* and *S100a9^-/-^* male mice as compared with *S100a9^+/+^* male mice, albeit significant differences were not observed (**Supplemental Figures 2B, C**). It should be noted that a significant increase in tubular Mac2 expression was similarly observed in S100a9-deficient mice (males and females), although the significance of this finding remains unknown.

### S100a9-Deficiency Does Not Alter Levels of HMGB1 and RAGE

While S100a9-deficiency drives disease development in male NZBWF1 mice, several of the humoral readouts tested suggested an immunostimulatory function of S100a9 in female animals. S100a9 has been shown to bind to multiple inflammatory molecules including high-mobility group box 1 (HMGB1) and receptor for advanced glycation end products (RAGE). We therefore tested levels of serum HMGB1 and soluble RAGE in male and female *S100a9^+/+^*, *S100a9^+/-^* and *S100a9^-/-^* NZBWF1 mice at 4-5 months of age. Neither molecule was affected by expression of S100a9 and neither differed between male and female mice (**Supplemental Figure 3**), suggesting that these pathways were not accountable for the different outcome of S100a9-deficiency in males and females.

### S100a9-Deficiency Drives Neutrophil Accumulation in Spleens of Male NZBWF1 Mice, but No Change in BAFF Levels

**Figure 7:**
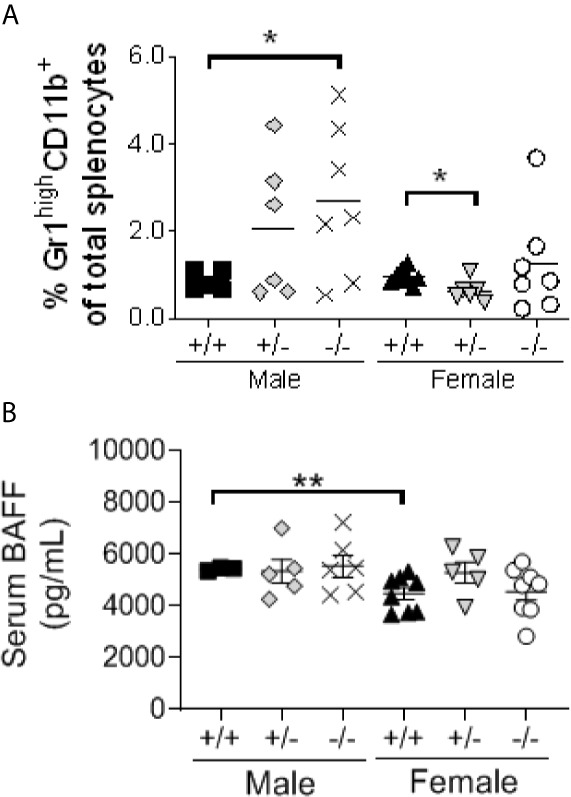
Male S100a9-deficient BWF1 mice express elevated levels of mature neutrophils, but unaltered levels of BAFF. **(A)** Levels of Gr1^high^CC11b^+^SSC^high^ mature neutrophils was measured in spleens of male and female *S100a9* ^+/+^, *S100a9* ^+/-^ and *S100a9* ^-/-^ mice by flow cytometry. **(B)** Serum BAFF levels were measured by ELISA on serum from 4 month old mice. Each dot represent one mouse. Shown is Mean ± SEM. *p < 0.05; **p < 0.01. One-way ANOVA: p < 0.05 **(A)**, p = 0.14 **(B)**.

Further understanding the mechanism behind lupus-like disease development in S100a9-deficient male NZBWF1 mice is imperative for the identification of *de novo* molecular or cellular therapeutic targets. Previously, a population of B cell activating factor (BAFF)-producing B helper neutrophils (N_BH_) was described located in the marginal zone in secondary lymphoid organs in response to inflammation, either induced or chronic as in SLE (49). We therefore tested levels of mature neutrophils (Gr1^high^CD11b^+^SSC^high^) in spleens of S100a9-deficient and –sufficient, male and female NZBWF1 mice along with systemic levels of BAFF (**Figures 7A, B**). While levels of Gr1^high^CD11b^+^SSC^high^ cells were ~ 3 fold higher in *S100a9^-/-^* male NZBWF1 mice than in *S100a9^+/+^* males and all female mice, we found no differences in levels of serum BAFF, suggesting that another mechanism may be responsible for driving lupus-like disease in male S100a9-deficient mice.

### S100a9-Deficiency Results in Elevated ISG Signature in Male NZBWF1 Mice

**Figure 8:**
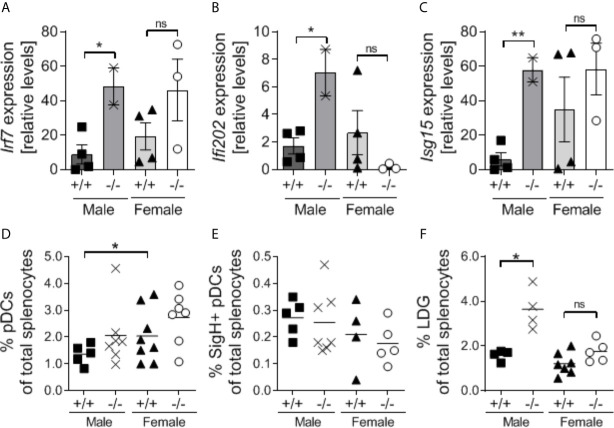
Male S100a9-deficient BWF1 mice express elevated expression of interferon-stimulated genes and increased levels of low-density granulocytes. **(A–C)** Real-time RT-PCR was performed on total splenocytes obtained from a subset of *S100a9* ^+/+^ and *S100a9* ^-/-^ male and female BWF1 mice at the time of harvest (≥ 7 months of age) and levels of *Ifi202* **(A)**, *Irf7* **(B)**, and *Isg15* **(C)** were determined. Levels of total pDCs **(D)**, SigH^+^ pDCs **(E)**, and Low-density granulocytes (LDGs) **(F)**, were determined by flow cytometry. Each dot represent one mouse. Shown is Mean ± SEM. *p < 0.05; **p < 0.01, ns, not statistically significant. One-way ANOVA: p < 0.05 **(A)**, p = 0.08 **(B)**, p = 0.07 **(C)**, p = 0.09 **(D)**, p = 0.67 **(E)**, p = 0.0001 **(F)**.

It has been suggested that S100a9, granulocytic MDSCs (Gr1^high^CD11b^+^) and the interferon-related factor 7 (IRF7) exist in an auto-regulatory loop (50). Since IRF7 is essential for type I interferon production and type I interferons are known to drive lupus-like disease in NZBWF1 mice (51, 52), we tested transcript levels of *Irf7* and interferon-stimulated genes *Ifi202* and *Isg15* in a small subset of S100a9-sufficient and -deficient male and female NZBWF1 mice. Interestingly, all three genes were significantly upregulated in *S100a9^-/-^* male NZBWF1 mice as compared to *S100a9^+/+^* male NZBWF1 mice (p < 0.05-0.01) (**Figures 8A–C**). In contrast, neither gene was upregulated in *S100a9^-/-^* female NZBWF1 mice as compared with *S100a9* ^+/+^ females. Both *Irf7* and *Isg15* displayed slightly increased levels in WT females as compared to WT males. In lupus, type I interferons have been shown to be produced by either SiglecH^+^ plasmacytoid dendritic cells (pDCs) or low density granulocytes (LDGs) (53, 54). To evaluate if levels of these cell subsets were affected by S100a9-deficiency and could account for the suspected increased levels of type I interferons, we analyzed splenic frequencies of both cell subsets. We found no differences in the levels of SigH^+^ pDCs between *S100a9* ^+/+^ and *S100a9* ^-/-^ mice of either sex, despite elevated levels of total pDCs in female *S100a9^+/+^* mice as compared with male *S100a9^+/+^* mice (**Figures 8D, E**). In contrast, levels of total Gr1^+^CD11b^+^SSC^low^ LDGs were significantly elevated in male S100a9-deficient NZBWF1 mice (p < 0.05), but not in female S100a9-deficient mice (p = 0.12)(**Figure 8F**). Further analysis of LDGs in NZBWF1 mice identified elevated *Ifna* transcripts in both Ly6C^high^ (Gr1^low^) and Ly6C^low^ (Gr1^high^) LDG subsets, although differences only reached statistical significance in the Ly6C^high^CD11b^+^SSC^low^ population (**Supplemental Figure 4**).

## Discussion

Lupus, along with many other autoimmune disorders, is more prevalent in females than in males. While this discrepancy has been extensively investigated from the point of view that the immune system of females is hyperactive and therefore more likely to drive autoimmunity, only a few studies have evaluated why otherwise genetically predisposed males fail to develop disease. We previously reported that lupus-prone male NZBWF1 mice exhibited elevated levels of immunosuppressive neutrophil-like cells (Gr1^high^CD11b^+^ granulocytic MDSCs) and that depletion of these cells stimulated ANA production and IgG-IC deposition within the kidney glomeruli of lupus-prone male NZBWF1 mice (14). While we showed that the corresponding female MDSCs were immunosuppressive *via* the production of ROS/NOS only before puberty (at 4 weeks of age), male MDSCs remained immunosuppressive until at least 16 weeks of age (14). Here we show that MDSCs from male NZBWF1 mice utilize in part S100a9 as a mechanism of suppression, as cells from S100a9^-/-^ male NZBWF1 mice were unable to inhibit cytokine-driven B cell differentiation *in vitro*, control antibody responses to T-dependent NP-CGG antigen, and most importantly, control lupus-like disease development. As such, B and T cell hyperactivation, ANA production, IgG-IC deposition and complement C’3 fixation in kidney glomeruli, as well as renal damage, were all significantly enhanced in S100a9-deficient NZBWF1 male mice, but largely unaffected female S100a9-deficient NZBWF1 mice.

We previously suggested that the inflammatory milieu in lupus-prone female NZBWF1 mice may drive the maturation of MDSCs into pro-inflammatory neutrophils and/or macrophages, as previously shown (55). Subsequent studies by others showed that MDSC-like cells from female NZBWF1 mice were indeed effectively eliminated in a process involving IFNα, IFNγ, IL-6 and ROS production (15, 49, 56). Oppositely, depletion of neutrophils (including MDSCs) resulted in increased IFNγ production by NK cells (57). In lupus, both IFNα and IFNγ have been suggested as drivers of disease. For example, blocking or elimination of the IFNγ receptor was previously shown to significantly reduce disease development in female NZBWF1 mice (58), showing that IFNγ contributes to disease. Interestingly, NK cell driven IFNγ production is induced by pDC-derived IFNα, and inhibition of IFNα or the IFNα/β-receptor has similarly been shown to ameliorate lupus-like disease (52, 59). Finally, MDSCs were shown to be upregulated in the absence of Irf7 in a tumor model (50). Irf7 is induced downstream of both Toll-like receptor 7 (TLR7), TLR9, and type I interferon receptor ligation, all key molecules in mouse lupus pathogenesis. Thus, it is likely that in the presence of high levels of IFNα, as seen in some lupus patients and many mouse models of lupus, Irf7 is upregulated and MDSCs downregulated. Interestingly, Irf7 binding sites have been identified in the S100a9 promoter (50), further supporting an autoregulatory loop between S100a9, MDSCs and Irf7. In our study, we found that *Irf7* mRNA was upregulated in male *S100a9^-/-^* NZBWF1 mice as compared to their S100a9-sufficient littermates. We suggest that *Irf7* mRNA is upregulated due to elevated levels of IFNα, which is supported by the concomitant upregulation of two other ISGs: *Isg15* and *Ifi202*, and the accumulation of *Ifna*-expressing LDGs in S100a9^-/-^ NZBWF1 males. Thus, in this system, a lack of S100a9 interferes with the function of MDSCs, which in turn may lead to an accumulation of IFNα-producing LDGs, driving T and B cell activation, autoantibody production and end-organ inflammation and damage.

Surprisingly, female S100a9-deficient NZBWF1 mice displayed some evidence for reduced humoral disease as determined by reduced levels of activated T and B cells, reduced GC formation and diminished splenomegaly, although no differences in renal parameters were observed. In that regard, S100a9 has been found to possess both pro- and anti-inflammatory properties under different conditions. For example, some studies indicate that S100a8/a9 possess pro-inflammatory functions consistent with the complex being a TLR4 ligand (60, 61), while other evidence has suggested an immunoprotective role (33, 34, 39, 62). The two conclusions are not mutually exclusive and may depend on one of several variables such as the timing of investigation, whether inflammation is infectious or sterile, which cytokines are produced, which organ system is involved, and the sex of the animal. More studies are needed to determine if the latter is indeed involved, as the sex of the animals studied was not consistently reported in several of these studies, and no study compared the response in males and females. Both lupus patients and female NZBWF1 mice express elevated levels of several pro-inflammatory cytokines including IFNα, TNFα, IFNγ that may act to differentiate immunosuppressive MDSCs into proinflammatory MDSCs. In fact, we observed previously that Ly6C^high^CD11b^+^ MDSCs became immunostimulatory in female NZBWF1 mice as the mice aged (14). Here we observed elevated levels of Gr1^high^CD11b^+^SSC^high^ neutrophilic cells in male S100a9-deficient mice, suggesting that these cells may be involved in the pathogenesis. Others have found that a subset of B helper neutrophils capable of producing BAFF was significantly increased in autoimmune mice and people with autoimmune diseases (49) however, we found no evidence for differential levels of BAFF in S100a9-sufficient and –deficient NBWF1 mice, and thus a potential pathogenic mechanism by mature neutrophils in S100a9-deficient male mice remains unknown. Finally, it should also be mentioned that the conformation of S100a9-contaning multimers can affect the function of the complex. As such, it has been shown that S100a8/S100a9 heterodimers can drive an inflammatory immune response *via* binding to TLR4/Mdm2 receptors, while (S100a8/a9)_2_ tetramers are unable to bind TLR4 and thus unable to drive inflammation (63). Whether S100a9-containing complexes are different in male and female NZBWF1 mice, and how such complex formation is regulated, remains to be fully determined.

Besides transcriptional regulation as discussed above, post-translational modifications have been implicated in the function of S100a8 and/or S100a9. Post-translational modifications, including S-nitrosylation, S-gluthathionylation and phosphorylation, have been proposed to be differentially associated with pro- and anti-inflammatory functions of S100a9 (64–66). Recent studies have identified dysregulated miR-146a-5p and miR-155-5p miRNAs as drivers of phosphorylated S100a8/a9 heterodimers and the production of proinflammatory cytokines in female rheumatoid arthritis patients (67). Both of these microRNAs have also been found to be dysregulated in SLE patients (68, 69). No study has yet evaluated if this observation correlates with the presence of phosphorylated, proinflammatory S100a9 in lupus, although phosphorylated S100a9 has been found upregulated in SLE patients (70) and to exert pro-inflammatory functions (64). Oppositely, S-nitrosylated S100a8 (and S100a9 to a lesser extend) has been proposed to inhibit leukocyte-endothelial cell interactions and leukocyte extravasation due to regulation of CD11b expression (66). Interestingly, S-nitrosylation is estrogen-dependent in endothelial cells (71), although whether estrogens have a similar effect on S100a8 and S100a9 in neutrophils remains unknown. Finally, the long-non-coding RNA Hotairm1 was found to drive the immunosuppressive function of MDSCs *via* its binding to S100a9 which functionally blocked secretion and led to an accumulation of the protein in the nucleus (72). Further studies are needed to identify the subcellular localization and post-translational modification status of S100a9 (and S100a8) in male and female lupus-prone NZBWF1 mice and SLE patients.

In summary, S100a9 is expressed and secreted by myeloid cells, including MDSCs, and exerts an immunosuppressive role in male, but not female, lupus-prone NZBWF1 mice. S100a9-expressing MDSCs functionally suppress B cell differentiation *in vitro*, B and T cell activation *in vivo*, and spontaneous autoantibody production. Interestingly, S100a9 may play a different role in female mice, as S100a9-deficiency results in slightly reduced disease patterns. Thus, as future studies evaluate the effect(s) of therapeutically targeting S100a9 or S100a8/S100a9 in various diseases, studying responses in both males and females will be of utmost importance to elucidate if such therapy will be equally effective in both sexes.

## Data Availability Statement

The original contributions presented in the study are included in the article/**Supplementary Material**. Further inquiries can be directed to the corresponding author.

## Ethics Statement

The animal study was reviewed and approved by Cleveland Clinic Foundation Animal Research Committee guidelines and the Institutional Animal Care and Use Committee of the Lerner Research Institute of the Cleveland Clinic Foundation.

## Author Contributions

LD developed the mouse model, helped with mouse harvests, ran flow cytometry, did immunization assays, and ran the NP-ELISA. AA helped with mouse harvests, ran S100a8/a9 ELISA, and performed immunofluorescence staining on kidney tissue. AD performed Hep2G assay, dsDNA ELISA, ISH imaging, and helped write the manuscript. MP did anti-chromatin, anti-histone, IgG, and IgM ELISA, helped with mouse harvests and flow cytometry, and analyzed immunostainings of the kidneys. EK performed HMGB1, RAGE, and BAFF ELISA, ISG RT-PCR, performed and analyzed all GC immunofluorescence staining on spleen tissue, did select flow analyses, and helped revise the manuscript. ND performed trichrome staining, and S100a8 and S100a9 immunohistochemistry. AW helped with ISG RT-PCR. AK did all B cell differentiation assays. NS performed S100a4/a8/a9 RT-PCR on sorted cells. LL did flow analyses. TV generated S100a9-deficient mice and helped revise the manuscript. TJ designed the study, supervised all analyses, mentored students, and wrote the manuscript.

## Funding

This study was supported in part by the Department of Defense Grant #W81XWH-11-1-0667 (TJ).

## Conflict of Interest

The authors declare that the research was conducted in the absence of any commercial or financial relationships that could be construed as a potential conflict of interest.
