# Supplementary figures and images for: S100a9 Protects Male Lupus-Prone NZBWF1 Mice From Disease Development
Source: Front Immunol. 2021 Jun 17;12:681503. doi: 10.3389/fimmu.2021.681503 (PMC8248531; doi:10.3389/fimmu.2021.681503)

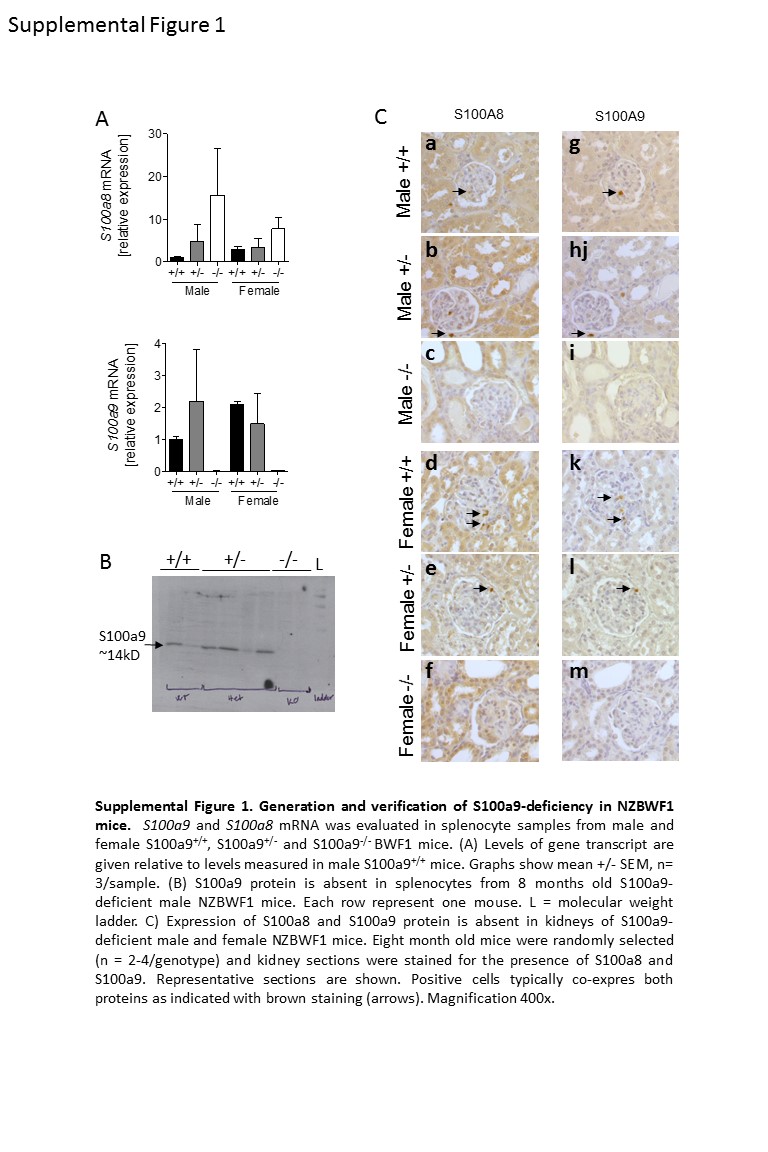

Supplement: Supplementary file 1 [file Image_1.jpg]

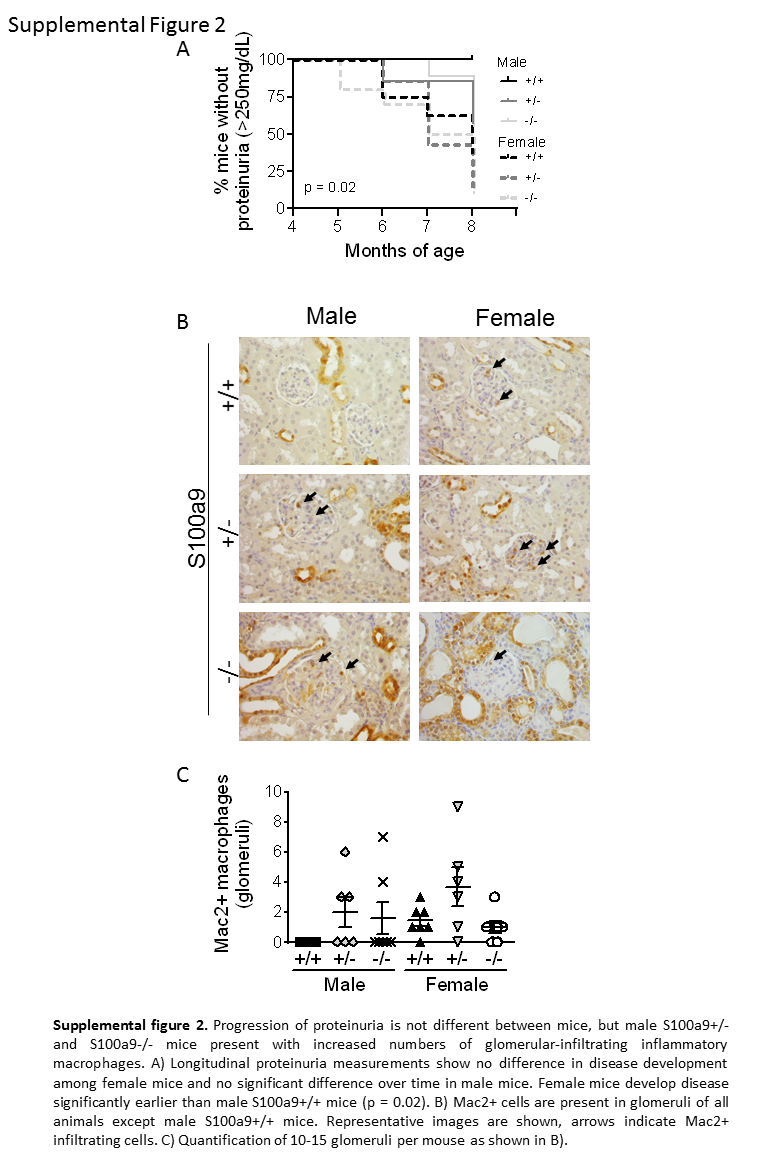

Supplement: Supplementary file 2 [file Image_2.tif]

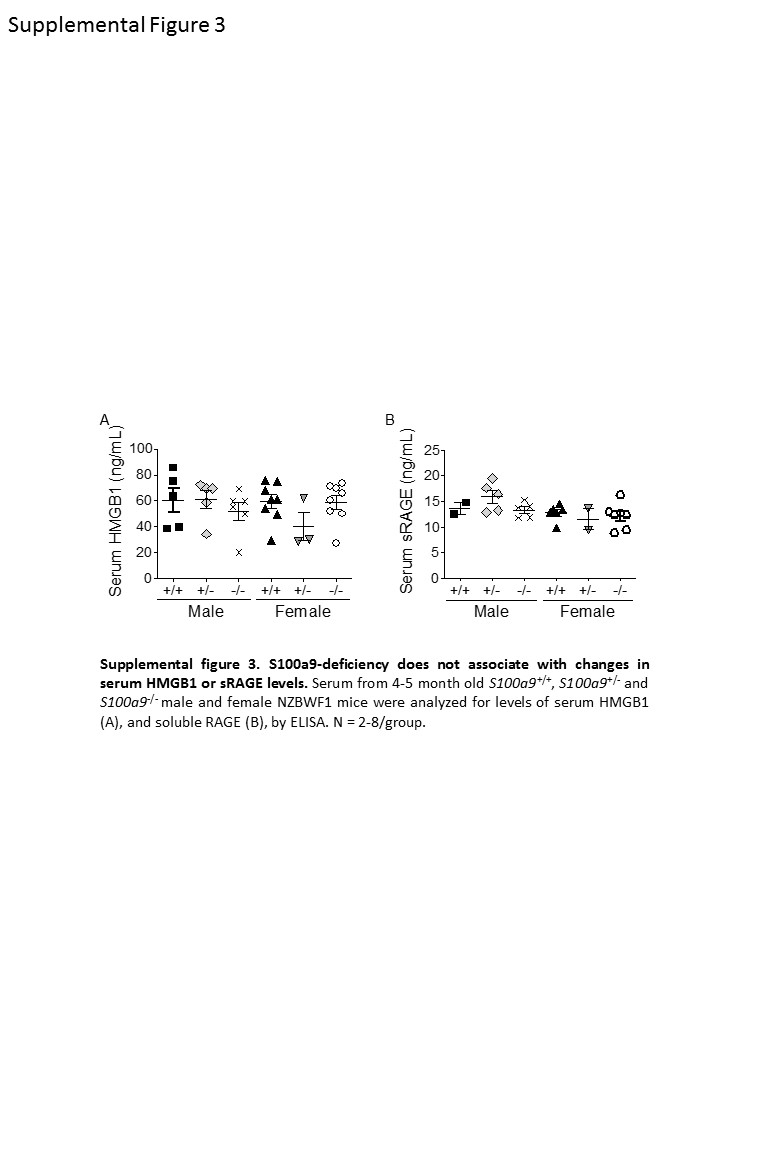

Supplement: Supplementary file 3 [file Image_3.jpg]

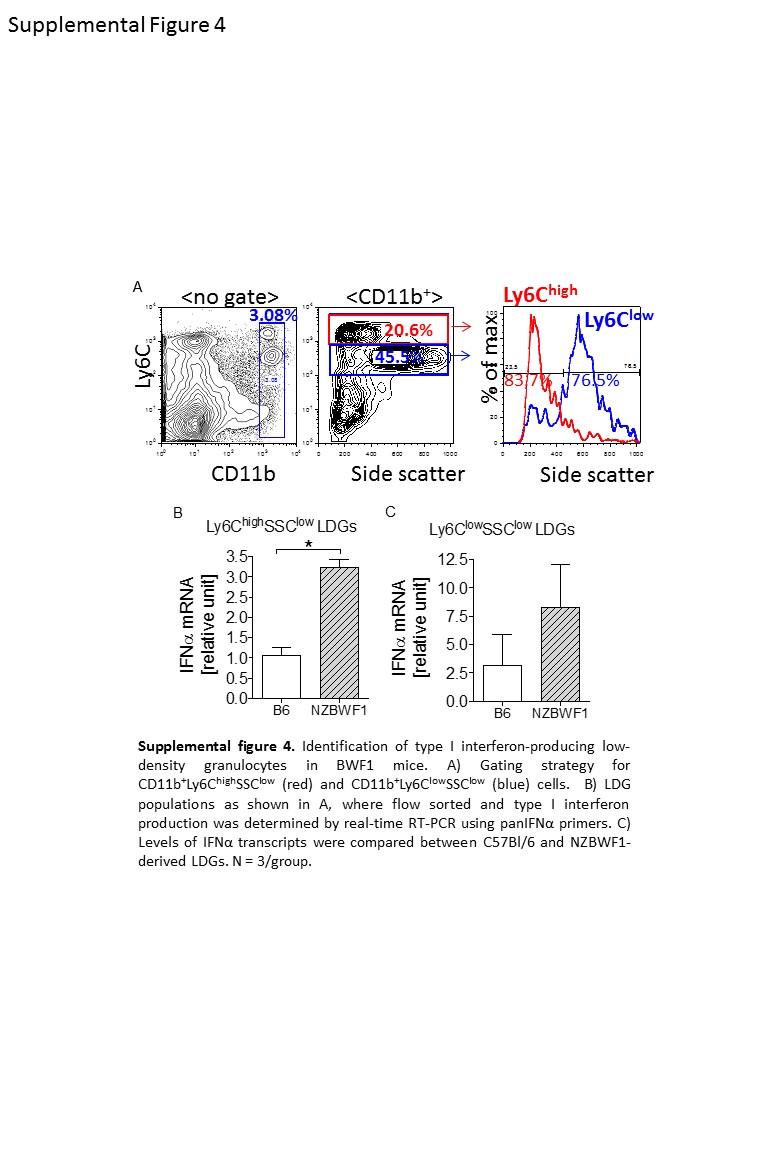

Supplement: Supplementary file 4 [file Image_4.jpg]
